# Analysis of multipacting threshold sensitivity to the random distributions of the secondary electron yield parameters

**DOI:** 10.1038/s41598-024-51289-z

**Published:** 2024-01-08

**Authors:** Firozeh Kazemi, Maryam Mostajeran, Gennady Romanov

**Affiliations:** 1 Faculty of Physics, Yazd University, P.O. Box 89195-741, Yazd, Iran; 2 Fermi National Accelerator Laboratory, Batavia, IL 60510 USA

**Keywords:** Physics, Plasma physics, Statistics

## Abstract

The way multipacting develops, depends strongly on the secondary emission property of the surface material. The knowledge of secondary electron yield is crucial for accurate prediction of the multipacting threshold. Variations in secondary electron yield parameters from experimental measurements create uncertainty, stemming from handling and surface preparation, and these uncertainties significantly affect multipacting threshold predictions. Despite their significance, the previous studies on the multipacting phenomenon did not adequately address the effect of an assumed random distribution of the secondary emission parameters on the multipacting threshold. Therefore, this paper aims to provide a comprehensive statistical study on how the different random distributions of the secondary emission parameters and, as a result, the uncertainty in the secondary electron yield affect multipacting thresholds. We focus on three commonly used distributions, namely uniform, normal, and truncated normal distributions, to define the uncertainty of random inputs. We use the chaos polynomial expansion method to determine how much each of the random parameters contributes to the multipacting threshold uncertainty. Additionally, we calculate Sobol sensitivity indices to evaluate the impact of the individual parameters or groups of parameters on the model outputs and study how different random distributions of these parameters affected the Sobol index results.

## Introduction

High-power radio frequency (RF) devices operating under vacuum conditions are potentially susceptible to the occurrence of multipacting breakdown. Multipacting is an electromagnetic phenomenon primarily caused by secondary electrons in particle accelerators, microwave tubes, antennas, RF windows, and space equipment^1^.

It is necessary to implement the practical measures to ensure the safe operation of RF devices and prevent multipacting discharge. Methods like third harmonic detection or phase vacuum detection can identify the multipacting discharge^2,3^. These methods can play an important role in assessing the risk of multipacting and ensuring proper device design. However, due to the high costs of these experimental methods, the theoretical approaches^4–6^ and the numerical studies^7^ are predominantly used to predict the multipacting threshold and achieve optimal design of RF devices. Therefore, the accuracy of multipacting threshold prediction may significantly affect the performance of RF devices. For accurate multipacting threshold prediction, it is necessary to consider the unavoidable secondary emission yield (SEY) variations associated with measuring the SEY data from the experimental samples with an uncertain history of handling and surface preparation. SEY is defined as the ratio of the number of secondary electrons (N_sec_) to the incident or primary electrons (N_inc_) on the material surface:

1 $$SEY = \frac{{N_{\sec } }}{{N_{inc} }}.$$

**Figure 1:**
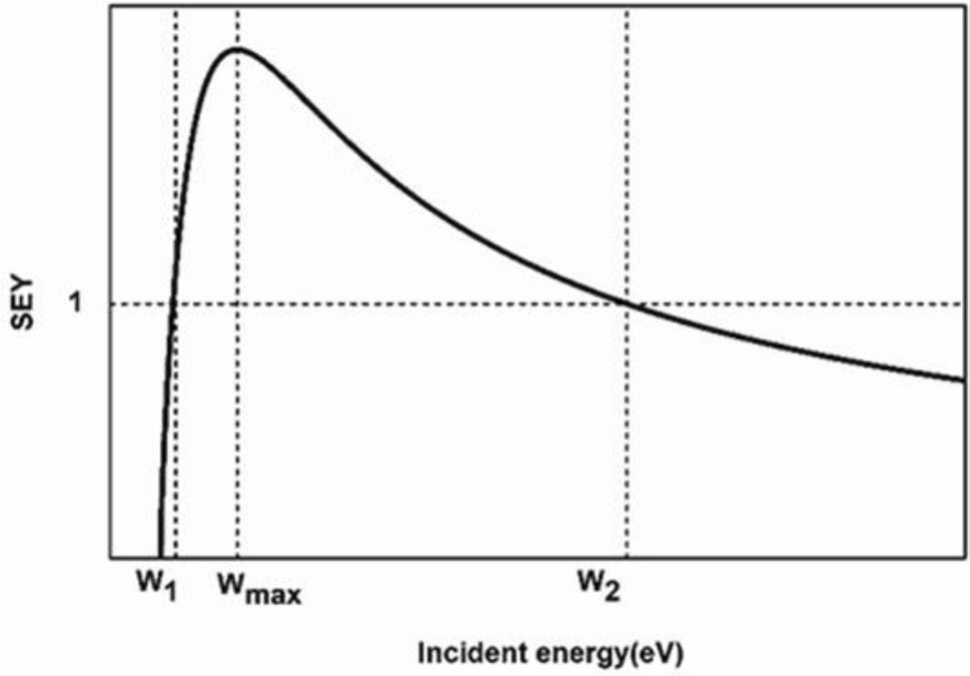
The behaviour of the secondary electron yield (SEY) with respect to the incident electron energy.

Figure 1 shows the general behaviour of SEY as a function of the incident electron energy. There are several models of secondary emission, such as the Furman and Pivi^8^, Vaughan^9^, and Sombrin models^10^, to describe the SEY versus the incident electron energy.

The SEY curve is determined by four key parameters: W_1_ and W_2_, which represent the crossover energy values at which SEY = 1, the maximum SEY value SEY_max_, and W_max_, the energy that corresponds to SEY_max_.

Measuring the SEY as a function of incident energy is a surface-sensitive process. The measured SEY values are highly influenced by the treatment of material surfaces prior to entering the vacuum. Despite the efforts to maintain repeatability in many SEY measurements, the significant uncontrolled variables during these measurements can lead to discrepancies between reported and actual values. Factors such as exposure to air, ambient temperature, cleanliness of measurement equipment, and other variables can create deviations in SEY measurements^11^, and it is important to consider these variations when analyzing and interpreting SEY data. For example, the experimental observations have shown that exposure to air and subsequent oxidation can increase SEY_max_ of metals beyond their nominal values, typically ranging from 1 to 2, to values greater than 3^12^. Additionally, a surface layer of chemical pollution, which often forms after exposure to air, can also induce changes in SEY^13^. In other words, the reported SEY values have the uncertainties that contribute to the multipacting threshold uncertainty.

Uncertainty Quantification (UQ) methods such as generalized Polynomial Chaos expansion (gPC) can analyze how input uncertainties affect system performance and lead to more efficient RF system design and construction. In the RF systems the dielectrics are also widely used along with the metals and they are susceptible to multipacting occurrence due to the high SEY. The most typical example of dielectric usage is ceramic for vacuum RF windows^14–17^. On the disk type ceramic vacuum RF windows usually single-sided multipacting occurs. This article investigates the sensitivity of the threshold for single-sided multipacting on dielectrics. In this type of multipacting, the electrons are emitted from and collide with the same surface. For the mentioned disk, ceramic window, the single-side multipacting involves two fields: the RF electric field parallel to the dielectric surface (E_RF_), which accelerates the emitted electrons and the DC electric field perpendicular to the surface (E_DC_), which returns the electrons back to the surface of dielectric. Simulations of the single-side multipacting are often performed using a simplified flat surface model, as the results can be extrapolated to models that are more complex. A detailed description of the mechanism of single-sided multipacting can be found in Ref.^18^. In this paper, we use the PIC PARTICLE STUDIO module from the CST Studio Suit software to simulate the multipacting phenomenon using the simplified model. The initial conditions and physical parameters utilized in the simulations are presented in Table 1.

**Table 1:** Physical characteristics and initial conditions of the model.

Frequency	Initial energy	E_DC_	E_RF_
325 MHz	7.5 eV	12 kV/m	20.250 kV/m

So far, most of the multipacting studies focused on investigating multipacting mechanisms^19,20^, developing prediction techniques^21,22^, and mitigating this phenomenon using surface treatments^23^. However, limited analysis has examined how the uncertainties of the SEY parameters affect the multipacting threshold evaluation. In Ref.^24^, the impacts of uncertainties of W_1_ and SEY_max_ on the multipacting in SRF gun with triangular grooved surface were studied, the Furman model was used to describe the SEY. In Ref.^25^, the authors have examined the uncertainty of W_1_ and W_max_, and its impact on the multipacting threshold for dielectrics, employing the Sombrin model for SEY calculation. Both studies assumed a uniform distribution when considering uncertainties SEY parameters. This choice is often made in uncertainty quantification studies for simplicity. However, given the inherently random nature of these parameters, it is important to examine how different types of uncertainty distributions can affect the analysis of multipacting thresholds. Investigation of different distributions of uncertainty in SEY parameters can help us to make more accurate predictions of the multipacting threshold. In the current study, we consider the uncertainties in the three main parameters of SEY – W_1_, SEY_max_, and W_max_ – making it more comprehensive than previous studies that typically consider only one or two parameters. Table 2 provides the surface characteristics, including the material and values of SEY parameters of the proposed simple model.

**Table 2:** Surface characteristics of the model.

Material	W_1_ (eV)	W_max_ (eV)	SEY_max_
Teflon128	22	380	2.3

We employ the Sombrin model to calculate SEY as a function of incident energy, because the Sombrin model contains the three main SEY parameters (W_1_, SEY_max_ and W_max_) in its formulation. Additionally, the results from this model are close to the experimental data^26^. The table of the SEY data obtained with this model was imported into the CST PIC STUDIO to be used as imported secondary emission model.

In Sect. “Methodology” of this article, we will provide detailed description of the employed methodology, including an overview of the research process and the gPC approach. In Sect. “Calculating the multipacting threshold in CST software”, we will discuss an index for determining the multipacting threshold by the CST software. In Sect. “Results”, we will present the results of the univariate uncertainty, including investigation of uncertainty of W_1_, SEY_max_, and W_max_ with three different distributions. Furthermore, the results of the bivariable uncertainty, with three different distributions, will be also presented in this section. Bivariable uncertainty includes uncertainty of W_1_&W_max_, W_1_&SEY_max_, and SEYmax & W_max_. A variance based global sensitivity analysis is performed to determine which SEY parameters have the greatest influence on multipacting threshold, and the results of this analysis will be presented. Finally, the conclusion Sect. “Conclusion” provides a summary of the overall findings of the paper.

## Methodology

### Research process

#### Identifying distributions for SEY parameter uncertainties

The normal, uniform and truncated normal distributions are selected to model the uncertainties of the W_1_, SEY_max_ and W_max_ parameters.

#### Determining the random parameters

The three main SEY curve parameters—W_1_, SEY_max_ and W_max_—are considered as input random variables.

#### Defining the values of uncertainty

The uncertainty values are calculated using the relative standard deviation (σ_r_), defined as the ratio of the standard deviation (σ) to the mean of the parameter values (μ). Three values of uncertainty corresponding to σ_r_ = 5, 10, 30% are considered for each SEY parameter.

#### Investigation framework for analyzing multipacting

**Figure 2:**
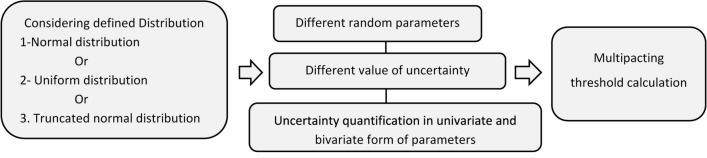
Research process in investigating the impact of random distribution of SEY parameters uncertainties on multipacting threshold uncertainty.

Multipacting simulations are performed for each random parameter separately for each uncertainty value and distribution. The gPC method is employed to calculate the  $\langle SEY\rangle$ function and multipacting threshold. Uncertainties in bivariable combinations (W_1_&W_max_, W_1_&SEY_max_ and SEY_max_ & W_max_) are also modeled as bivariate uncertainties. This process is represented in Fig. 2.

### The gPC method

The gPC technique was first introduced by Ghanem and Spanos for solving several engineering problems^27^. This method represents the output as a polynomial expansion in terms of orthogonal polynomials that are functions of the input parameters. The coefficients of the polynomial expansion are determined by projecting the output onto the basic functions of the orthogonal polynomials. The resulting polynomial approximation can be used to estimate the statistical quantities of the output, such as the mean and the variance, and to propagate the uncertainty of the input parameters to the output. Here, we describe the main steps of the gPC technique, which we applied to our modelling of the multipacting threshold uncertainty in this study.

#### Generation polynomial chaos

In the gPC method, the type of polynomial chaos depends on the probability distribution of the random parameters. Some polynomials according to the type of random distributions are shown in Table 3.

**Table 3:** Distributions types and orthogonal basis polynomial support ranges I^28^.

I	Distribution type orthogonal basis polynomial	Distribution orthogonal
(− ∞, ∞)	Hermit	Normal
[− 1, 1]	Legendre	Uniform
[0, 1]	Jacobi	Beta
(− 1, 1)	Laguerre	Exponential

In our study, we consider uniform, normal and truncated normal distribution for random parameters, so according to Table 3 we should use Legendre and Hermit polynomial respectively.

##### Normal distribution

A random variable X ~ N (μ, σ^2^) follows a normal distribution. This distribution is characterized by two parameters: the mean (μ), which represents the center of the distribution, and the variance (σ^2^), which determines the spread of the distribution. A random variable x with mean μ = 0 and standard deviation σ = 1 is said to be a standard normal random variable and is denoted as X ~ N (0, 1).

##### Uniform distribution

A random variable X ~ U [a, b] follows a uniform distribution. The parameters "a" and "b" represent the lower and upper boundaries of the defined interval, respectively, wherein the probability of each event occurring is equal. The uniform distribution is the simplest form of continuous probability distribution. The case where a = 0 and b = 1 is the standard uniform distribution.

##### Truncated normal distribution

The truncated normal distribution is a probability distribution obtained by constraining the normal distribution within a specific range and the values outside this specific range are truncated. The parameters of the truncated normal distribution are the same as those of the normal distribution: the mean (μ) and the variance (σ^2^), but with the additional specification of a range [a, b] for allowable values.

#### Generation the polynomial expansion

The basis of the gPC approach is to provide a polynomial surrogate for the computational model. In this context, the polynomial expansion represents the relationship between the system’s response (Y) and the independent input parameters $\vec{\xi }$ in an M-dimensional space. The polynomial expansion can be expressed as following:

2 $$Y = \sum\limits_{\mid \alpha \mid \, \le \,N} {{C_\alpha }{\varphi _\alpha }(\mathop \xi \limits^ \to )} .$$

where N is the degree of the polynomial expansion, α is the multi-index that indicate the degree of the polynomial in each of the input variables, C_α_ are the unknown coefficients to be determined and $\upphi_{\alpha } (\vec{\xi })$ represents the multivariate polynomial. In our investigation, the system response we are interested in, is the multipacting threshold, and we aim to approximate a function representation for this quantity.

#### Determination of expansion coefficients

The coefficients, C_α_, are determined by projecting the truncated expansion of Y on each basis polynomial and exploiting its orthogonality in the domain I:

3 $$C_{\alpha } = \langle \frac{1}{{\upphi_{\alpha } (\vec{\xi })\upphi_{\beta } (\vec{\xi })}}\rangle \int\limits_{I} {Y(\vec{\xi })} \upphi (\vec{\xi })D(\vec{\xi })d\vec{\xi }.$$

where $D\,(\vec{\xi })$ is the probability density function (PDF) of the random parameters. α and β are the multi-indices that indicate the order of the polynomial in each of the input variables. There are many methods for numerically multidimensional integration or quadrature, which is a classical problem.

In mathematics and numerical analysis, quadrature is an approximate method for computing integrals. Table 4 illustrates the comparison of integration techniques or quadrature and their respective integration points for three different random distributions.

**Table 4:** Comparison of integration techniques, polynomial quadrature and with integration for different random distribution.

Distribution/polynomial	Quadrature	$\langle \Phi_{\upalpha } (\xi )\Phi_{\upbeta } (\upxi )\rangle = \updelta_{\upalpha \upbeta } \upgamma_{\upalpha }$	n = integration points
Uniform/Legendre	Clenshaw–Curtis^29–31^	$\gamma_{\alpha } = \frac{2}{2\alpha + 1}$	2^N+1^
Normal/Hermit	Gussi-Hermit	$\gamma_{\alpha } = \sqrt \pi 2^{\alpha } \alpha \,!$	N + 1
Truncated normal/Hermit	Gussi-Hermit	$\gamma_{\alpha } = \sqrt \pi 2^{\alpha } \alpha \,!$	N + 1

The first column in Table 4 presents the types of distributions along with the corresponding polynomial quadrature method used. The “n = integration points” column represents the number of integration points utilized for each method. The second and third rows demonstrate the integration techniques applied to different distributions/polynomial. The Clenshaw-Curtis method is employed for the Uniform/Legendre distribution with 2^N+1^ integration points^29^.

Numerical integration methods, such as quadrature, are employed to calculate these coefficients.

For detailed information on integration techniques and their application to different random distributions, refer to Table 4.

##### Transformation for random variables

Since Y depends on the parameter X, a transformation must be determined and the standard random variable ξ_i_ is mapped onto the random variable X_i_. For example in our study, we interest in the deviation interval of W_1_, SEY_max_ and W_max_. We used the inverse transform method, which relies on the principle that continuous cumulative distribution functions (CDFs) are uniform in the interval [1, 0]^32^. Here in, Table 5, we provide the transformation equations, integration points, and ranges of parameter deviations for σ_r_ = 5%, 10%, and 30% of W_1_.

**Table 5:** Standard range of ξ and range of deviation for σ_r_ = 5%, 10%, 30% of W_1_ = 22 (eV).

Distribution type	Standard range of ξ_i_	Transformation equation	σ_r_ in W_1_	Deviation interval of W_1_ = 22 (eV)
Uniform	ξ_i_ ~ U[− 1, 1]	$X_{i} = \frac{b - a}{2}\xi_{i} + \frac{b + a}{2}$	5%	X_i_ ~ U[a = 20.9, μ = 22, b = 23.11]
			10%	X_i_ ~ U[a = 19.8, μ = 22, b = 24.2]
			30%	X_i_ ~ U[a = 15.4, μ = 22, b = 28.6]
Normal	ξ_i_ ~ N (μ = 0, σ = 1)	$X_{i} = \mu + \sigma \xi_{i}$	5%	X_i_ ~ N(μ = 22, σ = 1.1)
			10%	X_i_ ~ N(μ = 22, σ = 2.2)
			30%	X_i_ ~ N(μ = 22, σ = 6.6)
Truncated normal	ξ_i_ ~ N (μ = 0, σ = 1)	$X_{i} = \mu + \sigma \xi_{i}$	5%	X ~ N(μ = 22, σ = 1.1, a = 20.9, b = 23.11)
			10%	X_i_ ~ N(μ = 22, σ = 2.2, a = 19.8, b = 24.2)
			30%	X_i_ ~ N(μ = 22, σ = 6.6 a = 28.6, b = 28.6)

To compute the coefficients C_N_ in Eq. (3) and, consequently, approximate the Y, the deterministic model is evaluated at sparse grid nodes.

#### Accuracy assessment

For accuracy assessment of the gPC, a posteriori error estimate approach is used to calculate the relative error for the (N + 1)st order of the gPC. In this study, we estimated this relative error using expansion coefficients. The formula related to the relative error is provided in Appendix A.

#### Global sensitivity analysis (Sobol’s indices)

Sensitivity analysis assesses the influence of uncertain inputs parameters and interactions on the output variable (Y). A global, variance-based approach is valuable for customizing models by identifying inputs with minimal impact and quantifying the potential reduction in output uncertainty if these inputs were known. To achieve these objectives, Sobol introduce global, variance-based sensitivity indices^33^.

The first-order Sobol sensitivity index, also referred to as the main sensitivity index, quantifies the portion that X_i_ contributes directly (without interaction) to the total variance of the output V[Y]. It aids to the identification of uncertain inputs that could be more precisely evaluated, thus facilitating input prioritization. The index is defined by Eq. (4);

4 $$S_{i} = \frac{{V_{{X_{i} }} }}{V},$$

where V_Xi_ represents the variance associated with the f (X_i_) and V is the total output variance.

Second-order sensitivity indices represent the portion of variance resulting from X_i_ and X_j_ interaction, they are defined as Eq. (5);

5 $$S_{i,j} = \frac{{V_{{X_{i} ,X_{j} }} }}{V}.$$

V_Xi, Xj_ represents the variance associated with the Y (X_i_, X_j_).

More details about the steps of the gPC technique are given in the Appendix A.

## Calculating the multipacting threshold in CST software

The multipacting threshold refers to the combination of the system/model parameters at which multipacting discharge begins. In this study, the effects of space charge are not taken into account. Typically, in Multipacting simulations without space charge effects, the increase of the number of particles exhibits exponential behavior. However, there are instances where this assumption does not hold true; in such cases, the concept of the effective secondary electron yield is used as an index for the multipacting threshold. This parameter is not influenced by the specific manner in which the number of particles grows over time. As a result, it serves as a more robust and reliable indicator for Multipacting occurrence^34^.

By formal definition, the effective secondary electron yield $\langle SEY\rangle$ is the ratio of the average number of secondary particles emitted from the surface to the average number of incident particles. In the context of this study, $\langle SEY\rangle$ serves as an indicator of the multipacting threshold. Specifically:

$\langle SEY\rangle > 1$: implies the occurrence of multipacting.

$\langle SEY\rangle = 1$: signifies the onset of multipacting.

$\langle SEY\rangle < 1$: indicates the absence of multipacting.

In CST software, it is more convenient to define $\langle SEY\rangle$ via currents, i.e. SEY is calculated as:

6 $$\left\langle {{\text{SEY}}} \right\rangle = \frac{{{ }\left\langle {{\text{I}}_{{{\text{emission}}}} } \right\rangle }}{{{ }\left\langle {{\text{I}}_{{{\text{collision}}}} } \right\rangle }},$$

where $\left\langle {{\text{I}}_{{{\text{emission}}}} } \right\rangle$ and $\left\langle {{\text{I}}_{{{\text{collision}}}} } \right\rangle$ are the emission and collision currents averaged over the last three RF periods of the simulation time. The sufficiently long simulation time allows the system to reach a developed and stable multipacting process, which improve the accuracy and robustness of our results. The averaging in its turn mitigates noise and fluctuations. All the operations are performed within post-processing tool of the CST software.

With the E_DC_ value of 12 kV/m, we performed multipacting simulations across various E_RF_ ranges to determine the radio frequency field amplitude required to reach the multipacting threshold. However, it should be noted that the CST software does not provide sufficiently high precision for obtaining the value of $\langle SEY\rangle$ = 1 exactly. For the E_RF_ field amplitude of 20.250 kV/m, we considered the onset of multipacting to occur at $\langle SEY\rangle$ = 1.0065, accurate up to two decimal places.

**Figure 3:**
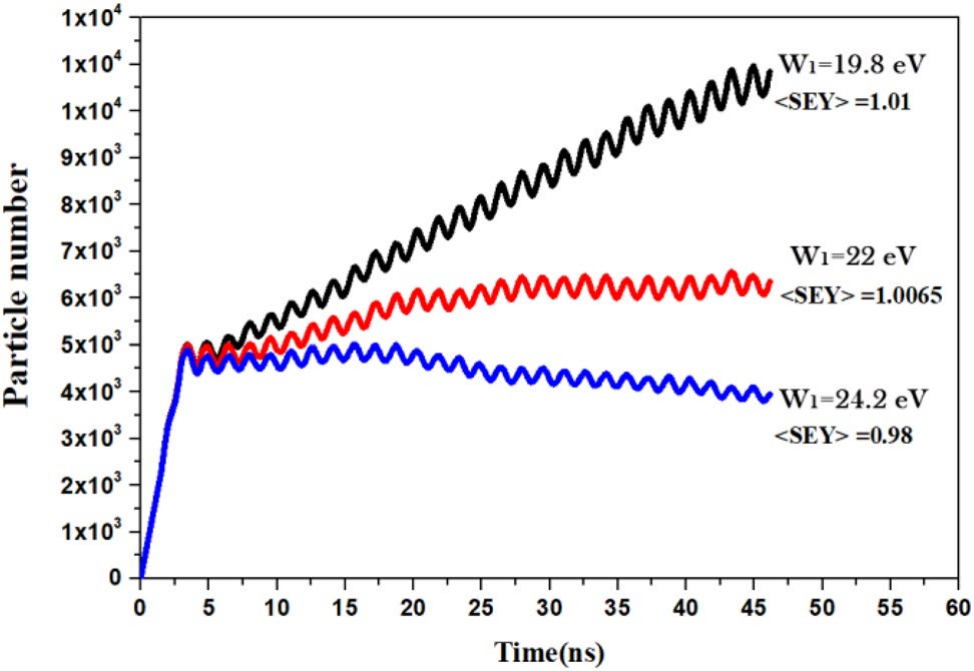
Particle number versus time.

In Fig. 3, we have plotted the particle numbers versus time for W_1_ = 22 eV and some deviations of this reference value of it. We observed that when the number of particles increases over time, the value of $\langle SEY\rangle$ increases to 1.01, and this indicates the occurrence of multipacting. Therefore, in this study, we considered the occurrence of multipacting when $\left\langle {{\text{SEY}}} \right\rangle \,\, \ge \,\,1.01$.

## Results

### Univariate uncertainty quantification results

#### Uncertainty of W_1_

First, we examined the effect of uncertainty of W_1_ (its nominal value is 22 eV) on $\langle SEY\rangle$, using the gPC method with three distributions: uniform, normal, and truncated normal. We modeled the uncertainty values by calculating the standard deviation quantity (σ_r_) as 5%, 10%, and 30%. In our analysis, we assumed constant values for W_max_ (380 eV) and SEY_max_ (2.3).

**Figure 4:**
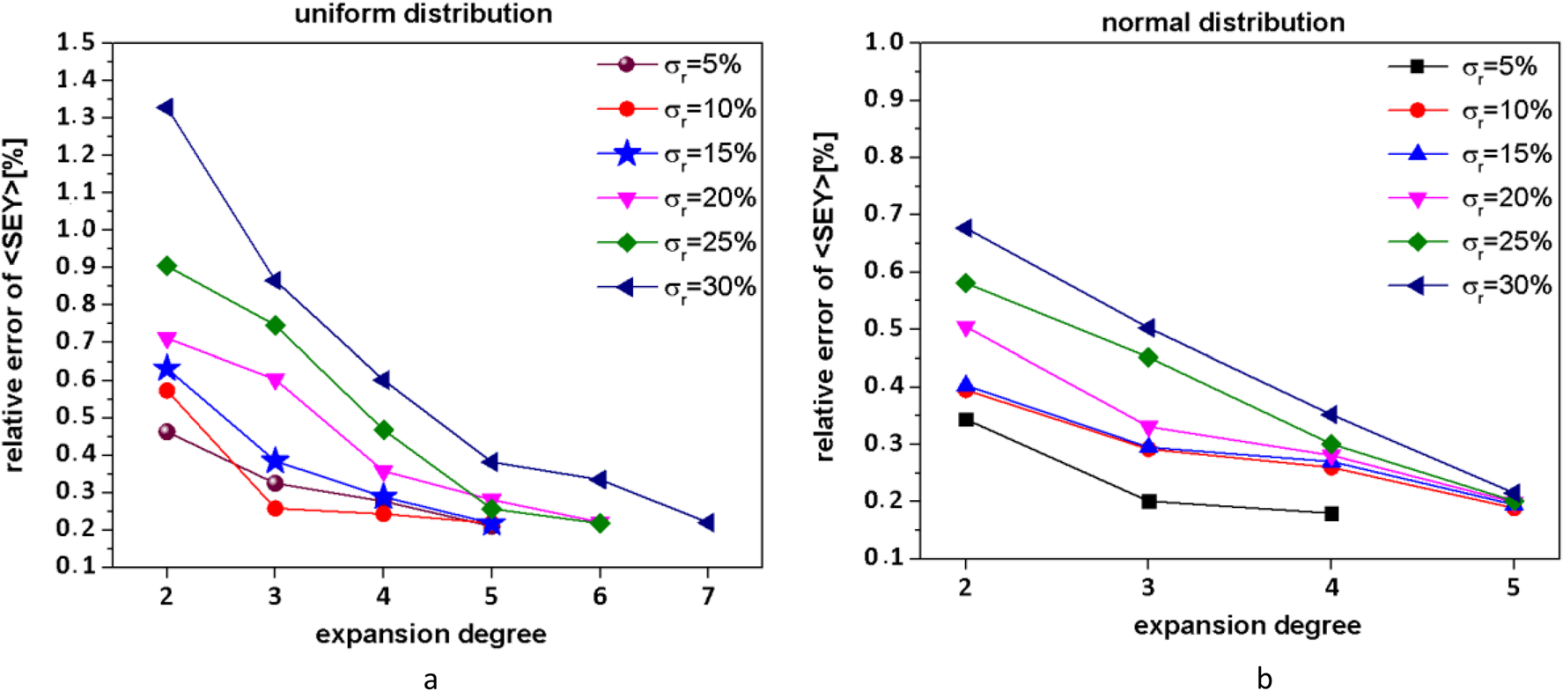
Relative errors versus expansion degrees, for inputs with, (**a**) uniform distribution by Clenshaw-Curtis quadrature, (**b**) normal distribution by Gussi-Hermit quadrature.

We extended the approximation of the function to the expansion degree for which the calculated relative error of  $\langle SEY\rangle$ becomes less than 0.25% as it was also employed in the reference^24^. In Fig. 4a and b, the relative error at different expansion degrees for normal and uniform distributions is illustrated respectively*.*

From the Fig. 4a and b, the relative error $\langle SEY\rangle$ for the uncertainty of 30% reduced below 0.25% for the 7th and 5th expansion degree for uniform and normal distribution respectively. Therefore, 7th and 5th expansion degrees have been chosen for subsequent evaluations.

The uncertainty modeling and calculations were performed using the Python programming language in conjunction with the CST software. The statistical quantities related to the calculated values of $\langle SEY\rangle$ for σ_r_ = 5, 10, 30% of W_1_ are presented in Table 6 including the following quantities:

- The expansion degree
- The average value of $\langle SEY\rangle$ is denoted as μ.
- The standard deviation is represented as σ.
- The variance is indicated as Var.
- The percentage change for μ of $\langle SEY\rangle$ compared to the multipacting threshold value is calculated as:
  7 $$\langle SEY\rangle_{error} = \left( {\frac{\mu - 1.0065}{{1.0065}}} \right) \times 100$$

**Table 6:** Statistical quantities of $\langle SEY\rangle$ for different σ_r_ of W_1_ with different distributions.

σ_r_ of W_1_	Distribution type	Expansion degree	μ of $\langle SEY\rangle$	$\langle SEY\rangle_{error}$	σ of $\langle SEY\rangle$	Var of $\langle SEY\rangle$
5%	Uniform	5	1.0069	0.03	0.0157	1.30E–04
	Normal	4	1.0064	0.02	0.0220	4.85E–04
	Truncated normal	4	1.0062	0.04	0.0116	1.34E–04
10%	Uniform	5	1.0070	0.04	0.0214	5.88E–04
	Normal	5	1.0087	0.21	0.0430	1.85E–03
	Truncated normal	5	1.0067	0.01	0.0242	5.85E–04
30%	Uniform	7	1.0270	2.03	0.0922	0.0056
	Normal	5	1.0383	3.15	0.1412	0.0199
	Truncated normal	5	1.0088	0.14	0.0468	0.0022

- The results in Table 6 indicate that the multipacting threshold (μ of $\langle SEY\rangle$) remains unchanged up to two decimal places in the cases of σ_r_ = 5, 10%, of W_1_ with any distribution. However, with an increase of the σ_r_ of W_1_ up 30%, the multipacting threshold changes for the normal and uniform distributions, while it remains unchanged for the truncated normal distribution.
- The dispersion of $\langle SEY\rangle$, that measured by σ and Var, is approximately equal for the uniform and truncated normal distributions of W_1_ with σ_r_ = 5, 10%. Moreover, this dispersion is lower compared to the normal distribution. With increased σ_r_ of W_1_ up to 30%, the dispersion of $\langle SEY\rangle$ became higher for the uniform distribution than the truncated normal distribution. However, the normal distribution still exhibits the highest values of σ and Var, indicating that it possesses the greatest inherent dispersion of $\langle SEY\rangle$.
- In the cases of σ_r_ = 30% of W_1_, $\langle SEY\rangle$ is significantly higher in the normal distribution than in the other two distributions.

We have shown comprehensive results, which include the evaluation of three additional levels of uncertainty (σr = 15%, 20%, 30%) of W_1_, in Table B1 in Appendix B.

#### Uncertainty of SEY_max_

In this section, we examine the impact of uncertainty of SEY_max_ = 2.3. Following the same approach used in the previous section, we keep W_max_ = 380 eV and W_1_ = 22 eV constant. The corresponding statistical quantities of $\langle SEY\rangle$ are presented in Table 7, for σ_r_ = 5, 10, 30% of SEY_max_.

**Table 7:** Statistical quantities of $\langle SEY\rangle$or different σ_r_ of SEY_max_ with different distributions.

σ_r_ of SEY_max_	Distribution type	Expansion degree	μ of $\langle SEY\rangle$	$\langle SEY\rangle_{error}$	σ of $\langle SEY\rangle$	Var of $\langle SEY\rangle$
5%	Uniform	5	1.0072	0.06	3.10E-04	5.59E–07
	Normal	3	1.0085	0.19	8.30E-04	2.30E–05
	Truncated normal	3	1.0082	0.16	2.84E-04	6.24E–06
10%	Uniform	6	1.0085	0.19	0.0013	1.78E–06
	Normal	4	1.0084	0.18	0.0065	4.17E–05
	Truncated normal	4	1.0077	0.11	0.0014	1.96E–06
30%	Uniform	7	1.0105	0.39	0.0020	4.19E–06
	Normal	4	1.0147	0.81	0.0266	7.06E–04
	Truncated normal	4	1.0110	0.44	0.0035	1.21E–05

- According to the Table 7, for σ_r_ = 5, 10 of SEY_max_, μ of $\langle SEY\rangle$ does not change significantly for any of the distributions. This implies the multipacting threshold remains unchanged at these lower uncertainty amounts. However, for σ_r_ = 30% of SEY_max_, μ of $\langle SEY\rangle$ for all three distributions increases, that indicates a change in the multipacting threshold at the higher uncertainty amounts (σ_r_ = 30%) of SEY_max_.
- σ and Var values for the normal distribution are higher compared to the uniform and truncated normal distributions for the all uncertainty amounts (σ_r_ = 5, 10, 30%).
- An important point is that the differences in results (in terms of μ, σ, Var of $\langle SEY\rangle$ and $\langle SEY\rangle$_error_) for the different distributions of the SEY_max_ are relatively smaller when compared to the differences observed for the distributions of W_1_ in the previous section.

A more comprehensive set of results is included in Appendix B, including the assessment of three additional uncertainty levels (σ_r_=15%, 20%, 30%) of parameters SEY_max_ in Table B2.

#### Uncertainty of W_max_

In this section, we investigate the impact of uncertainty of the W_max_ (its nominal value is 380 eV) on $\langle SEY\rangle$ with three different distributions. For this analysis, we keep the parameters SEY_max_ and W_1_ constant at 2.3 and 22 eV, respectively. Table 8 presents the statistical properties of $\langle SEY\rangle$ considering W_max_ uncertainties σ_r_ = 5, 10, 30%.

**Table 8:** Statistical quantities of $\langle SEY\rangle$ for different σ_r_ of W_max_ with different distributions.

σ_r_ of W_max_	Distribution type	Expansion degree	μ of $\langle SEY\rangle$	$\langle SEY\rangle_{error}$	σ of $\langle SEY\rangle$	Var of $\langle SEY\rangle$
5%	Uniform	3	1.0066	0.002	0.0011	6.03E–07
	Normal	3	1.0075	0.09	0.0027	7.07E–06
	Truncated normal	3	1.0062	0.04	6.89E-04	4.74E–07
10%	Uniform	4	1.0061	0.05	0.0012	1.23E–06
	Normal	3	1.0049	0.16	0.0026	6.80E–06
	Truncated normal	3	1.0048	0.17	2.45E-04	5.98E–08
30%	Uniform	5	1.0064	0.02	0.0026	4.46E–06
	Normal	4	1.0087	0.21	0.0029	8.25E–06
	Truncated normal	4	1.0062	0.04	9.29E-04	8.64E–07

- The data in Table 8 shows that μ of $\langle SEY\rangle$ and $\langle SEY\rangle$_error_ remain relatively similar across distributions for each different σ_r_ of W_max_, so it can be concluded that the uncertainty of W_max_, ranging from σ_r_ = 5% to 30%, does not significantly change the multipacting threshold. This implies that the W_max_ parameter has a minor impact on multipacting, making it the least effective parameter among those considered.
- However, normal distributions of these different σ_r,_ produce more dispersion of $\langle SEY\rangle$ as indicated by σ and Var.

#### Uncertainty propagating of $\langle SEY\rangle$ by univariate analyzes

**Figure 5:**
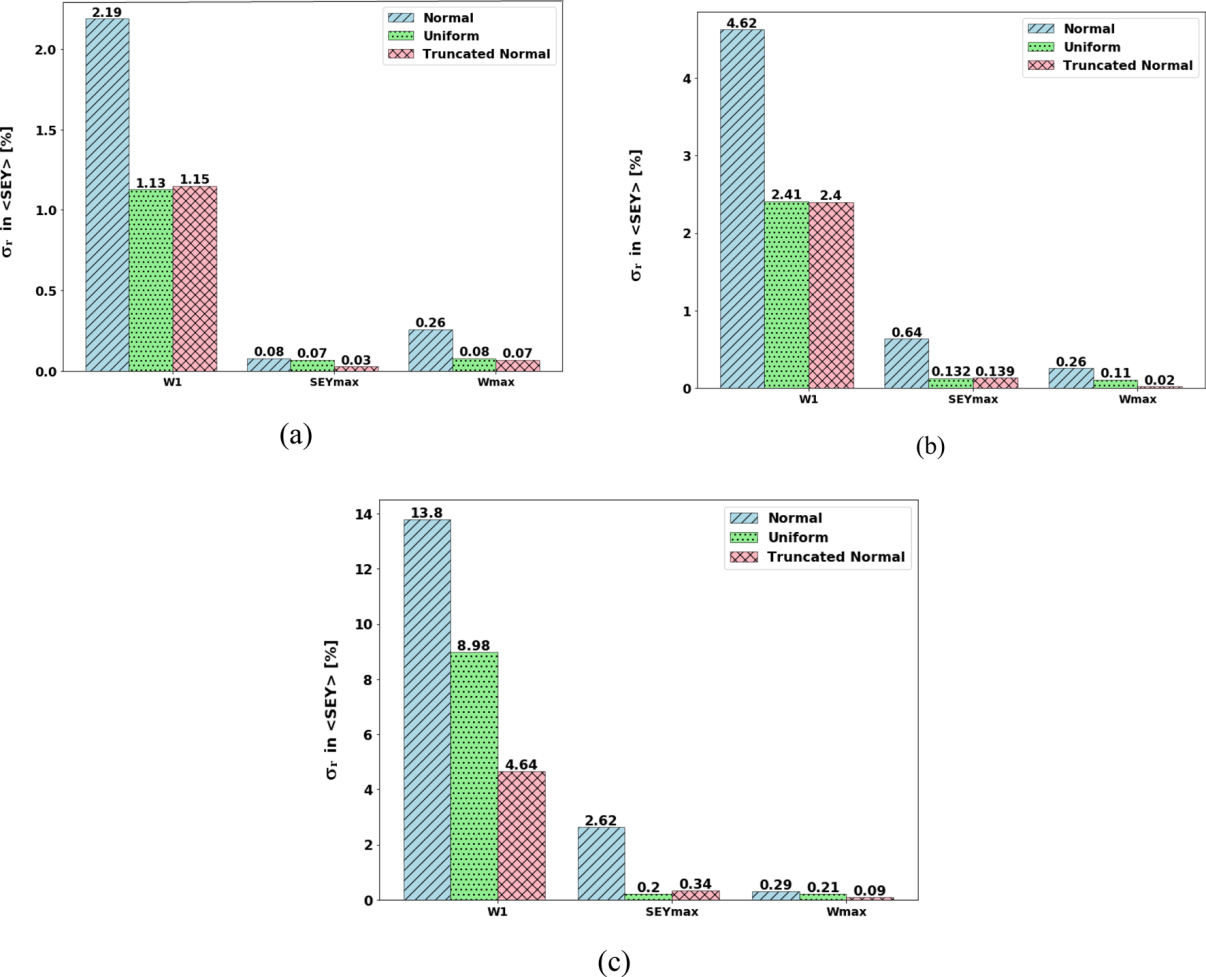
σ_r_ of $\langle SEY\rangle$, due to the σ_r_ of the input parameters with three different distributions (**a**) σ_r_ = 5% of the input parameters (**b**) σ_r_ = 10% of the input parameters (**c**) σ_r_ = 30% of the input parameters.

Figure 5 shows the uncertainty of $\langle SEY\rangle$ values (σ_r_ of $\langle SEY\rangle$) due to the uncertainties of three input parameters: W_1_, SEY_max_ and W_max_. The plots a, b and c represent three different σ_r_ = 5, 10, 30% respectively. Each block in the plots corresponds to an input parameter and contains 3 bars for the three distributions examined: normal, uniform and truncated normal distributions. The bars show the σ_r_ of $\langle SEY\rangle$ corresponding to each input parameter with different distributions.

- According to the Fig. 5, we can see that σ_r_ in W_1_ has a significantly larger contribution to the σ_r_ of $\langle SEY\rangle$ compared to SEY_max_ and W_max_, indicating that W_1_ is the most influential parameter.
- Fig. 5 also shows that the modelling of input parameter uncertainties (σ_r_) using different distributions (normal, uniform or truncated Normal) leads to different σ_r_ of $\langle SEY\rangle$.This means that the distribution type of the input parameter affects $\langle SEY\rangle$ uncertainty.
- For any of three input parameters and any σ_r_, the normal distribution causes higher uncertainty of $\langle SEY\rangle$ compared to the uniform and the truncated normal distributions.
- The Uniform and Truncated Normal distributions lead to relatively similar σ_r_ of $\langle SEY\rangle$.

### Bivariate uncertainty quantification results

This section investigates the impact of joint uncertainties in two input parameters simultaneously on the multipacting threshold. To conduct this analysis, we use bivariate gPC method. This allows for a more comprehensive analysis compared to considering parameter uncertainties individually. So in the following, the result of the joint uncertainties between W_max_ & W_1_, SEY_max_ & W_1_, and W_max_ & SEY_max_ on the $\langle SEY\rangle$ are provided respectively.

#### Uncertainty of W_1_ & W_max_

First, we assume SEYmax to be a constant value of 2.3. Using the bivariate gPC method, we calculate the $\langle SEY\rangle$ considering the σ_r_ = 5, 10, 30% of W_1_ and Wmax simultaneously. Subsequently, we compute the corresponding $\langle SEY\rangle$ values and present the statistical quantities in Table 9.

**Table 9:** Statistical quantities of $\langle SEY\rangle$ values for different σ_r_ in, W_1_ & W_max_ with different distribution.

σ_r_ in W_1_&W_max_	Distribution type	Expansion degree	μ of $\langle SEY\rangle$	$\langle SEY\rangle_{error}$	σ of $\langle SEY\rangle$	Var of $\langle SEY\rangle$
5%	Uniform	5	1.0066	0.001	1.48E–04	5.13E–04
	Normal	4	1.0063	0.03	0.0222	4.91E–04
	Truncated normal	4	1.0061	0.05	0.0121	1.47E–04
10%	Uniform	6	1.0078	0.12	0.0302	5.27E–04
	Normal	5	1.0119	0.53	0.0391	0.0015
	Truncated normal	5	1.0079	0.13	0.0215	4.63E–04
30%	Uniform	7	1.0301	2.34	0.0725	0.0053
	Normal	6	1.0398	3.30	0.1422	0.0202
	Truncated normal	6	1.0092	0.26	0.0758	5.75E–3

- According to Table 9 the multipacting threshold remains unchanged for σ_r_ = 5% with the three distributions for W_1_ & W_max_. For σ_r_ = 10% for two parameters, the multipacting threshold changes with normal distribution, but remains unchanged with uniform and truncated normal distributions. For σ_r_ = 30%, μ of $\langle SEY\rangle$ changes for both normal and uniform distributions of inputs, but remains unchanged for truncated normal distribution.
- The standard deviation (σ) and variance (Var) of $\langle SEY\rangle$ for truncated normal distribution of W_1_ &W_max_ are similar to that for the uniform distributions for the three different of σ_r_, and have higher values for normal distribution.

For two parameters, W_1_ & W_max_, the first and second order Sobol sensitivity indices were computed, taking into account σ_r_ = 30% applied simultaneously to the W_1_&W_max_ with the three different distributions.

**Figure 6:**
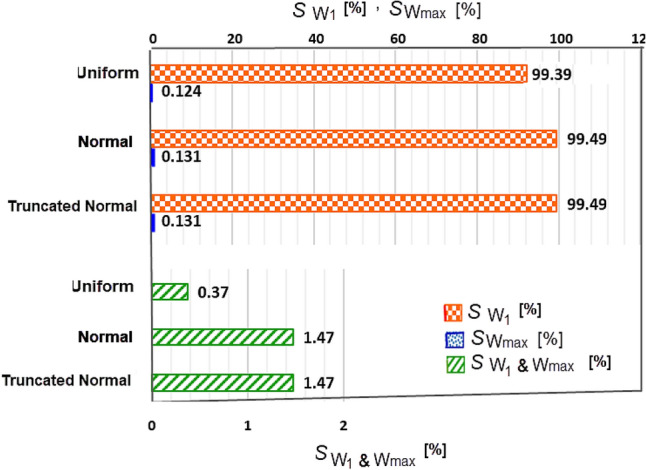
Result of Sobol indices for σ_r_ = 30% applied simultaneously to the W_1_&W_max_ with three distributions.

- According to Fig. 6, W_1_ has a significantly larger impact on $\langle SEY\rangle$ compared to the Wmax, ($S_{{W_{1} }} > > S_{{W_{\max } }}$). This indicates that deviations of W_1_ have a greater effect on the multipacting threshold than that of W_max_.
- For the normal and uniform distributions, *S*_W1_ values are almost the same, $S_{{W_{1} \,\left( {Uniform} \right)}} \, \cong \,\,S_{{W_{1} \,\left( {Normal} \right)}}$, suggesting that the variation of W_1_ with different distributions does not significantly affect $\langle SEY\rangle$
- Similarly, $S_{{_{{W_{\,\max } }} }}$ are also close for both distributions, i.e. $S_{{W_{\max \,} \left( {Uniform} \right)}} \, \cong \,\,S_{{W_{\max } \,\left( {Normal} \right)}}$. Small value of $S_{{_{{W_{\max } }} }}$ indicates that the variation of $\langle SEY\rangle$ is not strongly affected by the choice of distributions σ_r_ for W_1_ & W_max_.
- However, the second-order Sobol index ($S_{{w_{1} ,w_{\max } }}$), which shows the effects of interaction between two parameters on the $\langle SEY\rangle$, is higher for the normal distribution that for the uniform distribution This suggests that the normal distribution exhibits stronger effect of the parameters interaction on $\langle SEY\rangle$ than the uniform distributions.

#### Uncertainty of W_1_ & SEY_max_

Here we assume W_max_ to be constant at 380 eV. Using the bivariate gPC method, we calculate the $\langle SEY\rangle$ considering the combined uncertainties in W_1_ & SEY_max_ for different σ_r_ (See Table 10).

**Table 10:** Statistical quantities of $\langle SEY\rangle$ for different σr of W_1_& SEY_max_ with different distributions.

σ_r_ in W_1_,SEY_max_	Distribution type	Expansion degree	μ of $\langle SEY\rangle$	$\langle SEY\rangle_{error}$	σ of $\langle SEY\rangle$	Var of $\langle SEY\rangle$
5%	Uniform	3	1.0077	0.11	0.0118	1.39E–04
	Normal	3	1.0046	0.19	0.0234	5.47E–04
	Truncated normal	3	1.0061	0.05	0.0118	1.39E–04
10%	Uniform	5	1.0049	0.16	0.0250	6.24E–04
	Normal	4	1.0079	0.13	0.0402	0.0016
	Truncated normal	4	1.0082	0.16	0.0215	4.63E–04
30%	Uniform	5	1.0156	0.90	0.0743	0.0055
	Normal	5	1.0258	1.91	0.1237	0.0179
	Truncated normal	5	1.0125	0.59	0.0720	0.0052

- Based on the μ of $\langle SEY\rangle$ in the Table 10, it is observed that for σ_r_ = 5, 10% for W_1_ & W_max_, the $\langle SEY\rangle$ remains unchanged for any of the three distributions. However, as σ_r_ increases up to 30%, the $\langle SEY\rangle$ changes in the normal and truncated normal distributions while remaining unchanged in the uniform distribution.
- Furthermore, the standard deviation (σ) and variance (Var) of $\langle SEY\rangle$ for the truncated normal distribution of W_1_ & W_max_ are nearly similar to the uniform distribution and smaller than the normal distribution.

For two parameters, W_1_ & SEY_max_, the first and second order Sobol sensitivity indices are computed, taking into account σ_r_ = 30% with the three different distribution.

**Figure 7:**
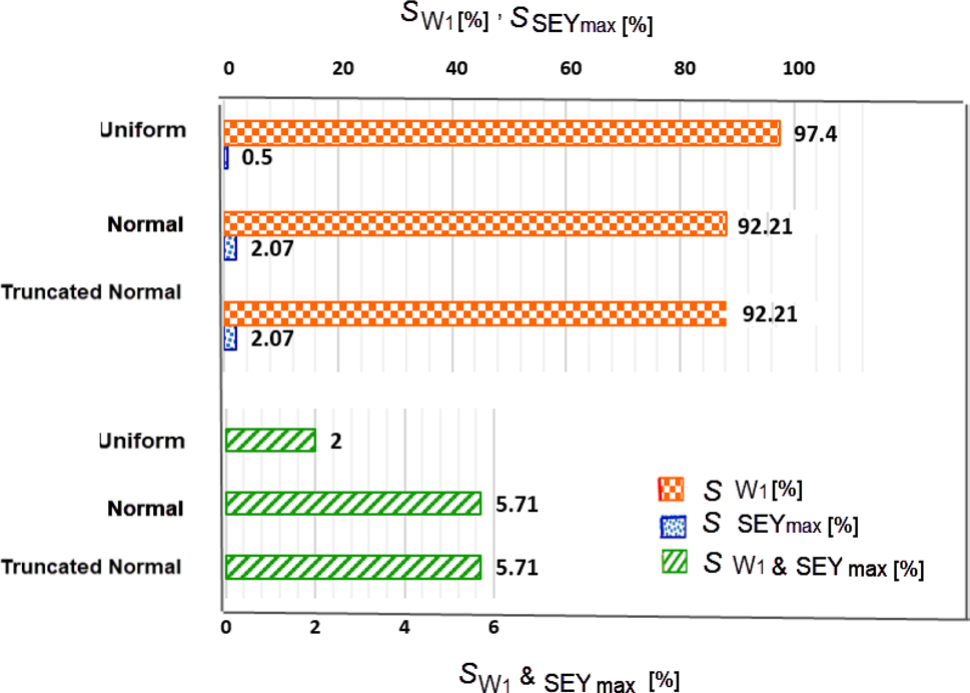
Result of Sobol indices for σ_r_ = 30% applied simultaneously to the W_1_&SEY_max_ with three distributions.

- According to Fig. 7, W_1_ has a significantly larger influence on $\langle SEY\rangle$ compared to the SEY_max_, ($S_{{W_{1} }} > S_{{SEY_{\max } }}$). This indicates that deviations in W_1_ have a greater effect on the multipacting threshold than deviations of SEY_max_.
- The results also show that for σ_r_ = 30% in W_1_&SEY_max_ parameters with a uniform distribution, $S_{{W_{1} }}$ is higher than normal distribution, $S_{{w_{1} \left( {Uniform} \right)}} \, > \,S_{{w_{1} \left( {Normal} \right)}}$, on the other hand $S_{{SEY_{\max } }}$ for the uniform distribution of σ_r_ in W_1_& SEY_max_, has a lower value than their normal distribution, $S_{{SEY_{\max } \left( {Uniform} \right)}} \, < \,S_{{SEY_{\max } \left( {Normal} \right)}}$. The second-order Sobol index of these two parameters ($S_{{_{{W_{1} ,SEY_{\max } }} }}$) for the normal distribution is higher than their uniform distribution,$S_{{W_{1} ,SEY_{\max } \left( {Normal} \right)}} \, > \,S_{{W_{1} ,SEY_{\max } \left( {Uniform} \right)}}$.

#### Uncertainty in SEY_max_ & W_max_

In this section we keep W_1_ = 22 eV constant and for various σ_r_, the bivariate gPC method is used to calculate the $\langle SEY\rangle$ function considering the joint uncertainties in SEY_max_ & W_max_. The corresponding $\langle SEY\rangle$ values are then computed, and the statistical quantities are presented in Table 11.

**Table 11:** Statistical quantities of $\langle SEY\rangle$ for different σr in SEYmax &Wmax with different distributions.

σ_r_ in SEY_max_, W_max_	Distribution type	Expansion degree	μ of $\langle SEY\rangle$	$\langle SEY\rangle_{error}$	σ of $\langle SEY\rangle$	Var of $\langle SEY\rangle$
5%	Uniform	5	1.0068	0.02	8.62E-04	7.44E–07
	Normal	3	1.0064	0.02	4.37E-04	1.91E–07
	Truncated normal	3	1.0060	0.06	0.00067	4.46E–07
10%	Uniform	4	1.0066	0.001	0.0022	4.69E–06
	Normal	4	1.0066	0.001	0.0039	1.57E–05
	Truncated normal	4	1.0060	0.06	0.0010	1.04E–06
30%	Uniform	6	1.0124	0.58	0.0020	1.44E–06
	Normal	5	1.0130	0.64	0.0052	2.68E–05
	Truncated normal	5	1.0104	0.38	0.0721	5.18E–03

- Based on the μ of $\langle SEY\rangle$ in the Table 11, it is observed that for σ_r_ = 5, 10% in SEY_max_ & W_max_, $\langle SEY\rangle$ remains unchanged for any of the three distributions. However, as the σ_r_ increases up to 30%, $\langle SEY\rangle$ changes with the three different distributions.
- Furthermore, the standard deviation (σ) and variance (Var) of $\langle SEY\rangle$ for the truncated normal distribution of SEY_max_ & W_max_ are nearly similar to the uniform distribution and smaller than their normal distribution.

For two parameters, SEY_max_ & W_max_, the first and second order of Sobol sensitivity indices are computed, taking into account σ_r_ = 30% with the three different distributions.

**Figure 8:**
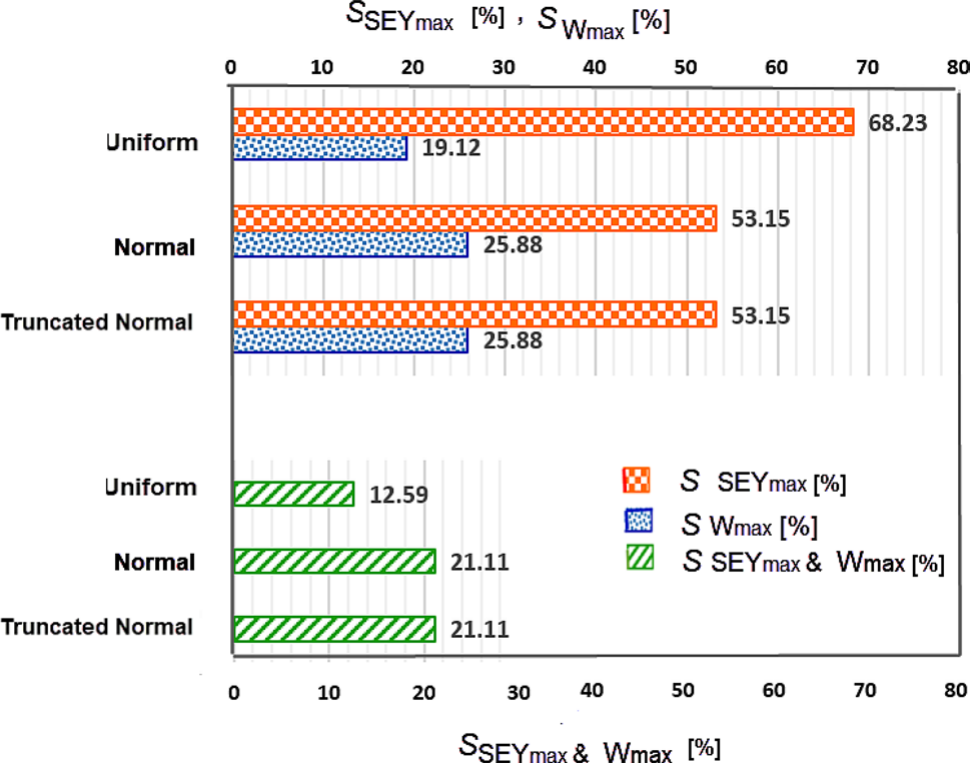
Result of Sobol indices for the σ_r_ = 30% applied simultaneously to the SEY_max_ & W_max_ with three different distributions.

- Results in Fig. 8 show that SEY_max_ has a larger influence on $\langle SEY\rangle$ compared to the W_max_, ($S_{{SEY_{\max } }} > S_{{W_{\max } }}$).
- The results show that for σ_r_ = 30% of W_1_&SEY_max_ parameters with the uniform distribution, $S_{{SEY_{\max } }}$ is higher than that with the normal distribution ($S_{{SEY_{\max } \left( {Uniform} \right)}} \, > \,S_{{W_{\max } \left( {Normal} \right)}}$).But on the other hand $S_{{W_{\max } }}$ for the uniform distribution of σ_r_ of W_1_& SEY_max_, has a lower value than that for their normal distribution ($S_{{SEY_{\max } \left( {Uniform} \right)}} \, < \,S_{{W_{\max } \left( {Normal} \right)}}$). The second-order Sobol index of these two parameters ($S_{{_{{SEY_{\max } ,W\max }} }}$) for the normal distribution is higher than that for the uniform distribution ($S_{{SEY_{\max } ,W_{\max } \left( {Normal} \right)}} \, > \,S_{{SEY_{\max } ,W_{\max } \left( {Uniform} \right)}}$).

#### Uncertainty propagating of $\langle SEY\rangle$ by bivariate analyzes

**Figure 9:**
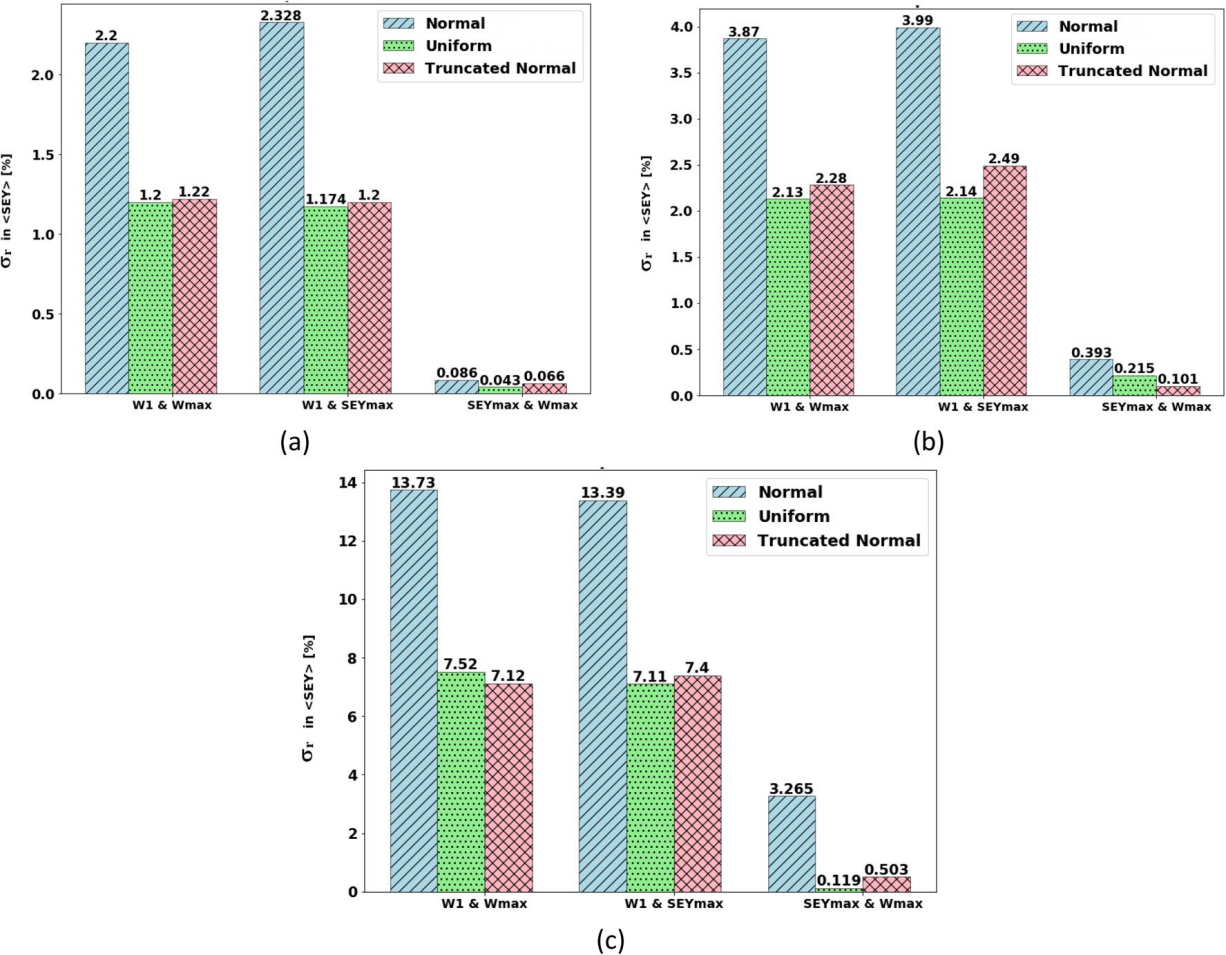
σ_r_ created in $\langle SEY\rangle$ (uncertainty created in $\langle SEY\rangle$ for the applying simultaneous σ_r_ in the input joint parameters (W_1_&W_max_), (W_1_&SEYmax) and (SEY_max_ & W_max_) with three different distributions (**a**) σ_r_ = 5% in inputs, (**b**) σ_r_ = 10% in inputs (**c**) σ_r_ = 30% in inputs.

Figure 9 shows the σ_r_ (uncertainty created) of $\langle SEY\rangle$ resulting from the simultaneous uncertainty of the input parameters (W_1_ & W_max_), (W_1_ & SEY_max_), and (SEY_max_ & W_max_) using three different distributions. The three values of uncertainty σ_r_ = 5%, 10%, and 30% were considered and the results are presented in the figures (a), (b), and (c), respectively.

Figure 9 shows that in the bivariate cases, with σ_r_ of the normal distribution for the combination of two parameters, σ_r_ of the multipacting threshold is higher. Additionally, these results indicate that the σ_r_ of $\langle SEY\rangle$ is nearly the same for the simultaneous changes in the pair of input parameters (W_1_&W_max_) and (W_1_&SEY_max_).

### Determining the allowable deviation range of SEY parameters

The findings presented in this article indicate that parameter W_1_ has the most significant impact on the multipactor threshold. The uncertainty contribution of this parameter is notably higher compared to the other two parameters. Furthermore, the Sobol sensitivity index for W_1_ is larger than that for the other parameters, suggesting that even a small variation of W_1_ can lead to a substantial deviation of the multipacting threshold. To determine the permissible range of SEY parameter deviations that would ensure no change of the multipacting threshold, we focused solely on exploring deviations of the W_1_ parameter. Specifically, we examined deviations of 15% and 20% in W_1_ to determine the extent of deviations at which multipacting does not occur.

The $\langle SEY\rangle$ for σ_r_ = 15% and 20% in W_1_ for the three different distributions are given in the Table 12.

**Table 12:** Statistical quantities of $\langle SEY\rangle$ for σ_r_ = 15, 20% in W_1_ with three different distributions.

σ_r_ in W_1_	Distribution type	μ of $\langle SEY\rangle$
15%	Uniform	1.0074
	Normal	1.0080
	Truncated normal	1.0063
20%	Uniform	1.014
	Normal	1.019
	Truncated normal	1.0082

$\langle SEY\rangle$ does not change for σ_r_ = 15% in W_1_, and it remains relatively constant for three different distributions. However, when σ_r_ increases to 20%, the μ of $\langle SEY\rangle$ for the uniform and normal distributions increase, leading to the multipacting occurrence. Therefore, we can conclude that deviations of up to 15% are acceptable for SEY parameter uncertainty.

## Conclusion

The aim of this study was to investigate the effect of different distributions of uncertainty (σ_r_) of the SEY parameters (W_1_, W_max_, and SEY_max_) on the uncertainty of the multipacting threshold i.e. $\langle SEY\rangle$.

According to the result, the different uncertainty distributions for the SEY parameters result in varying predictions for the multipacting threshold. The choice of distribution for the input parameters is important. For instance, when σ_r_ of W_1_ is equal to 30%, the normal distribution predicts the occurrence of multipacting, whereas both the uniform and the truncated normal distribution indicate its absence.

The investigation also reveals that the choice of random distribution for SEY parameters significantly affects the dispersion of $\langle SEY\rangle$. The standard deviation (σ) and variance (Var) consistently show higher dispersion for the normal distribution compared to the other two distributions. On the other hand, the truncated normal distribution of $\langle SEY\rangle$ results in lower values of $\langle SEY\rangle$ and less variability compared to variability of the uniform and normal distributions. This difference can be attributed to the inherent characteristics of the normal distribution, such as symmetry and tails extending to infinity, which contribute to larger deviations in the $\langle SEY\rangle$ values compared to the truncated normal and the uniform distributions.

The investigation reveals that the uncertainty of W_1_ has a more substantial impact on the uncertainty of $\langle SEY\rangle$ in comparison to the other two parameters, (W_max_ and SEY_max_). Furthermore, when examining the uncertainty of W_1_ together with either W_max_ or SEY_max_, their contributions to the overall uncertainty are almost equal. This observation can be explained by the significant influence of W_1_ on the outcome.

Based on the results obtained from Sobol’s sensitivity indices analysis, it can be concluded that the choice of different distributions significantly affects Sobol's sensitivity indices. The second-order sensitivity index assesses how the interaction between two SEY parameters influences the value of $\langle SEY\rangle$.

Comparing scenarios where parameters follow normal or uniform distributions, we observe that the second-order index yields higher values when normal distributions are used. It follows that parameters with normal distributions tend to exhibit stronger interactions, and this interaction has a greater effect on multipacting threshold variations. When uncertainty and errors in one parameter are reduced, uncertainty and errors in the other parameter may also be reduced. Controlling variations in the multipacting threshold thus becomes less costly and computationally intensive.

On the contrary, parameters with uniform distributions, which are more widely spread, might not interact so significantly. Therefore, the choice of parameter distribution becomes crucial, since it significantly affects our interpretation of Sobol indices. These indices, in turn, help us comprehend the relative impact of each parameter. When dealing with models involving multiple input parameters, the selection of parameter distribution becomes crucial as it directly affects the accuracy of the analysis and the results of Sobol indices.

Choosing an appropriate distribution for modeling uncertainties in a given model depends on the specific characteristics of the required data and the conditions of the case being studied. In this specific study, it appears that the truncated normal distribution is a suitable choice for modeling the uncertainty of SEY parameters. This choice is based on the following reasons:

- According to the reference^35^, the results with the truncated normal distribution for the parameters of a physical model are closer to the calculated theoretical values.
- In the truncated normal and normal distributions, the expansion coefficients and the approximation of the function are calculated using the Gauss-Hermit quadrature, which provides lower error compared to that of for the uniform distribution and Clenshaw-Curtis quadrature.
- The computation cost of expansion coefficients in the normal distribution is less than that in the uniform distribution. This is because the number of nodes in the Gaussi-Hermit quadrature method for the normal distribution is fewer than the nodes in the Clenshaw-Curtis quadrature method for the uniform distribution, consequently, the number of simulations for calculating < SEY > using the CST is reduced.
- The truncated normal distribution shows less change of $\langle SEY\rangle$ values and its dispersion, and does not have outlier data that cause incorrect prediction of the threshold compared to the normal distribution.

The consideration of the simultaneous uncertainty of three parameters (W_1_, SEY_max_ and W_max_) may provide more comprehensive results, and the correlations between them may be significant. However, undoubtedly, this comes at a higher computational cost. Therefore, the current study does not specifically address the three-variable case in order to manage computational resources efficiently.

### Supplementary Information

Supplementary Information.

## Data Availability

The data that supports the findings of this study are available within the article.
