# Supplementary material for: Analysis of multipacting threshold sensitivity to the random distributions of the secondary electron yield parameters
Source: Sci Rep. 2024 Jan 8;14:754. doi: 10.1038/s41598-024-51289-z (PMC10774443; doi:10.1038/s41598-024-51289-z)
Supplement: Supplementary file 1 — Supplementary Information. [file 41598_2024_51289_MOESM1_ESM.pdf]

Appendix A

The steps of the gPC method are described in more detail:

Generation the polynomial expansion

The basis of the gPC approach is to provide a polynomial surrogate for the computational model. In this context, the polynomial expansion represents the relationship between the system's response (Y) and the independent input parameters $\vec{\xi} = \{\xi_1 + \xi_2 + ...\xi_M\}$ in an M-dimensional space. The polynomial expansion can be expressed as following:

$$Y = \sum_{\alpha \in \chi(M,N)} C_\alpha \psi_\alpha(\vec{\xi}) \qquad \text{(A-1)}$$

where $\psi_\alpha(\vec{\xi})$ is the multivariate polynomial, M is the total number of input parameters, N is the degree of the polynomial expansion, and α is the multi-index that indicate the degree of the polynomial in each of the input variables:

$$\chi(M,N) = \alpha \in \left\{ N^M : \sum_{i=1}^{M} \alpha_i \le N \right\} \qquad \text{(A-2)}$$

C_α are the unknown coefficients to be determined. In our investigation, the system response we are interested in is the multipacting threshold, and we aim to approximate a function representation for this quantity. The total polynomial basis q during the surrogate modeling can be computed with the permutation formula:

$$q = \left(\frac{M+N}{N} \right) \qquad \text{(A-3)}$$

The multivariate polynomials are formed by the tensor products of the univariate orthogonal polynomials, which are as follows:

$$\psi_\alpha(\vec{\xi}) = \prod_{i=1}^{M} \Phi_{\alpha_i}^{(i)}(\xi_i) \qquad \text{(A-4)}$$

where $\Phi_{\alpha_i}^{(i)}$ is the univariate orthogonal polynomial in the ith variable of degree α_i[1].

Determination of expansion coefficients

The coefficients C_j are determined by projecting the truncated expansion of Y on each basis polynomial and exploiting its orthogonality in the domain I,

$$C_j = \langle \frac{1}{\psi_\alpha(\vec{\xi})\psi_\beta(\vec{\xi})} \rangle \int_I Y(\vec{\xi})\psi(\vec{\xi})D(\vec{\xi})d\vec{\xi}, \qquad \text{(A-5)}$$

Where $D(\vec{\xi})$ is the probability density function (PDF) of the random parameters. α and β are the multi-indices that indicate the degree of the polynomial in each of the input variables. There are many

methods for numerically multidimensional integration or quadrature, which is a classical problem. Here is a generic illustration of numerical integration methods:

$$C_n = \frac{\sum_{i=1}^{n} Y(\xi_i)\psi_i(\xi_i)\omega(\xi_i)}{\langle \psi_\alpha(\vec{\xi})\psi_\beta(\vec{\xi})\rangle}; \quad \text{(A-6)}$$

where ξ (i), $\omega(\xi_i)$ are the integration points and the weights respectively, and n is the number of integration points. Y (ξ_i) is the value of Y in the integration point ξ_i.

In mathematics and numerical analysis, quadrature is an approximate method for computing integrals. **Error! Reference source not found.** illustrates the comparison of integration techniques or quadrature and their respective integration points for three different random distributions.

To compute the coefficients C_N in Eq. (A-5) and, consequently, approximate the quantity $Y\left((X_1(\xi_1),.....X_M(\xi_M)\right)$, the deterministic model must be evaluated at the nodes $t^{(i)} = (t_1^{(i)},.....t_M^{(i)})$ of the sparse grid, where i range from 1 to n, representing the number of nodes. The numerical evaluation of the numerator in Eq. (A-5) can then be expressed as Eq. (A-7);

$$C_i \approx \sum_{i=1}^{n} Y\left(X_1(t_M^{(i)}),.....X_M(t_M^{(i)})\right) \times \psi_j(t^{(i)})\omega^{(i)}; \quad \text{(A-7)}$$

Where $\omega^{(i)}$ is the product of the corresponding weights. Once the ranges of deviations for the random parameter W_1 are determined, the CST software is executed for each value within these ranges using python code. The $\langle SEY\rangle$ is calculated for each random parameter, and the resulting $\langle SEY\rangle$ are saved in a text file. These results will used to calculate the expansion coefficients in Equation (A-7).

Calculation of the statistical output

This approximate expansion of the function can be used to estimate statistical moments of the output, such as the mean and the variance, and to transfer the uncertainty of the input parameters to the output.

Accuracy assessment

Accuracy of the gPC is determined by comparing the approximation with the actual output of the model. The accuracy depends on the choice of orthogonal polynomials and the number of basic functions used in the expansion. In this study, we use a posteriori error estimation method to determine the relative error for the (N+1)st degree of the gPC, given by Eq .(A-8) as in[2] :

$$\text{relative error} = \frac{\sqrt{\sum_{\alpha\in\chi(M,N+1)} \left|C_{\alpha_{N+1}} - C_{\alpha_N}\right|^2}}{\sqrt{\sum_{\alpha\in\chi(M,N+1)} \left|C_{\alpha_{N+1}}\right|^2}}. \quad \text{(A-8)}$$

Where C_{α_N} is the expansion coefficient of the Nth degree and $C_{\alpha_{N+1}}$ is the expansion coefficient of the (N +1) th degree.

Global sensitivity analysis (Sobol's indices)

Sensitivity analysis assesses the influence of uncertain inputs parameters and interactions on the output variable (Y). A global, variance-based approach is valuable for customizing models by identifying inputs with minimal impact and quantifying the potential reduction in output uncertainty if these inputs were known. To achieve these objectives, Sobol introduce global, variance-based sensitivity indices.
The first-order Sobol sensitivity index, also referred to as the main sensitivity index, quantifies the portion that X_i contributes directly (without interaction) to the total variance of the output V[Y]. It aids to the identification of uncertain inputs that could be more precisely evaluated, thus facilitating input prioritization. The index is defined by Eq. (A-9);

$$S_i = \frac{V_{X_i}\left[E\left[Y(\vec{X}) | X_i\right]\right]}{V\left[Y(\vec{X})\right]} = \frac{\sum_{\alpha\in\chi(1,N)} C_\alpha^2 \gamma_\alpha}{\sum_{\alpha\in\chi(M,N)} C_\alpha^2 \gamma_\alpha}; \qquad \delta_{\alpha\beta}\gamma_\alpha = \langle \psi_\alpha(\xi)\psi_\beta(\xi)\rangle . \tag{A-9}$$

Where V_{Xi} represents the variance associated with the f (X_i), the term $E\left[Y(\vec{X}) | X_i\right]$ represents the conditional expectation, which signifies the anticipated value of the f(X) given a specific value of the uncertain input Xi. Parameter γ_α is given for each integration method in Table 3.
Second-order sensitivity indices represent the portion of variance V[Y] resulting from X_i and X_j interaction, they are defined as Eq. (A-10);

$$S_{i,j} = \frac{V_{X_i,X_j}\left[E\left[Y(\vec{X}) | X_i, X_j\right]\right]}{V\left[Y(\vec{X})\right]} - S_i - S_j = \frac{\sum_{\alpha\in\chi(2,N)} C_\alpha^2 \gamma_\alpha}{\gamma_\alpha}. \tag{A-10}$$

$V_{Xi,\, Xj}$ represents the variance associated with the Y (X_i, X_j).

In the following, we calculate the relative error of <SEY> for each degree of the gPC expansion for different uncertainty levels (5%,….30%). We presented the results for parameter W_1 considering both normal and uniform distributions in Table A1.

Table A1: the gPC expansion degrees for different levels of uncertainty of W_1

σ_r of W1	Expansion degree	Relative Error[%] of <SEY> (Uniform distribution) (Clenshaw-Curtis quadrature)	Relative Error [%] of <SEY> (Normal distribution) (Gussi-Hermit quadrature)
5%	2	0.363	0.244
	3	0.325	0.201
	4	0.277	0.117
	5	0.210	-
**10%**	2	0.573	0.395
	3	0.258	0.272
	4	0.226	0.255
	5	0.219	0.189
**15%**	2	0.632	0.403
	3	0.480	0.295
	4	0.314	0.265
	5	0.218	0.190
**20%**	2	0.712	0.505
	3	0.603	0.331
	4	0.558	0.286
	5	0.401	0.199
	6	0.221	-
**25%**	2	0.905	0.581
	3	0.747	0.452
	4	0.568	0.301
	5	0.457	0.201
	6	0.218	-
**30%**	2	1.329	0.677
	3	0.766	0.503
	4	0.601	0.298
	5	0.482	0.215
	6	0.335	-
	7	0.22	-

Based on Table A1, the expansion degree used for the gPC at which the relative error falls below 0.25%, increased with the level of uncertainty .For the uncertainty of 5%, 10% and 15%, the 5th degree of expansion was used, and for the case of 20%, 25% and 30%, the 6th and 7th degrees of expansion were used. Furthermore, the obtained relative error of <SEY> using the Gussi-Hermit quadrature (normal distribution) is lower than that with the Clenshaw-Curtis quadrature (uniform distribution) used.

Appendix B

The statistical quantities related to the calculated values of <SEY> for different levels of uncertainties of σ_r = 5, 10, 15, 20, 25, 30% of W_1 are presented in Table B1

Table B1: Statistical quantities of $\langle SEY \rangle$ for different σ_r of W_1 with different distributions

σ_r of W_1	Distribution Type	μ of $\langle SEY \rangle$	$\langle SEY \rangle_{error}$	σ of $\langle SEY \rangle$	Var of $\langle SEY \rangle$
**5%**	Uniform	1.0069	0.03	0.0157	1.30E-04
	Normal	1.0064	0.02	0.0220	4.85E-04
	Truncated Normal	1.0062	0.04	0.0116	1.34E-04
**10%**	Uniform	1.0070	0.04	0.0214	5.88E-04
	Normal	1.0087	0.21	0.0430	1.85E-03
	Truncated Normal	1.0067	0.01	0.0242	5.85E-04
**15%**	Uniform	1.0074	0.08	0.0314	6.23E-04
	Normal	1.0080	0.14	0.0630	2.120E-03
	Truncated Normal	1.0063	0.03	0.0300	5.97E-04
**20%**	Uniform	1.0142	0.76	0.0722	0.0006
	Normal	1.0193	1.27	0.1012	0.0103
	Truncated Normal	1.0072	0.063	0.0368	0.0008
**25%**	Uniform	1.0160	0.94	0.0850	0.0011
	Normal	1.0266	1.99	0.1212	0.0123
	Truncated Normal	1.0079	0.13	0.0401	0.0012
**30%**	Uniform	1.0270	2.03	0.0922	0.0056
	Normal	1.0383	3.15	0.1412	0.0199
	Truncated Normal	1.0088	0.14	0.0468	0.0022

The results of Table B1 indicate the following:
For the uncertainty of 15%, similar to 5% and 10%, the multipacting threshold did not change for any of the random distributions. However, for the uncertainties of 20% and 25% of W_1, as well as the case of 30%, the multipacting threshold changed by one decimal place for two normal and uniform distributions, while for the truncated normal distribution <SEY> remained unchanged.
Table B2 includes statistical values related to <SEY> for the different uncertainty levels of SEY_{max}.

Table B2: Statistical quantities of $\langle SEY \rangle$ for different σ_r of SEY_{max} with different distributions

σ_r of SEY_{max}	Distribution Type	μ of $\langle SEY \rangle$	$\langle SEY \rangle_{error}$	σ of $\langle SEY \rangle$	Var of $\langle SEY \rangle$
**5%**	Uniform	1.0072	0.06	3.10E-04	5.59E-07
	Normal	1.0085	0.19	8.30E-04	2.30E-05
	Truncated Normal	1.0082	0.16	2.84E-04	6.24E-06
**10%**	Uniform	1.0085	0.19	0.0013	1.78E-06
	Normal	1.0084	0.18	0.0065	4.17E-05
	Truncated Normal	1.0077	0.11	0.0014	1.96E-06
**15%**	Uniform	1.0088	0.22	0.0014	5.03e-07
	Normal	1.0094	0.28	0.0030	2.07e-05
	Truncated Normal	1.0080	0.14	0.0025	5.61e-06
**20%**	Uniform	1.0092	0.26	0.0028	1.60e-06
	Normal	1.0096	0.30	0.0092	3.75e-05
	Truncated Normal	1.0081	0.15	0.0028	1.76e-06
**25%**	Uniform	1.0118	0.52	0.0050	1.19e-06
	Normal	1.0126	0.60	0.0112	2.45e-05
	Truncated Normal	1.0109	0.43	0.0041	1.58e-06
**30%**	Uniform	1.0105	0.39	0.0020	4.19E-06
	Normal	1.0147	0.81	0.0266	7.06E-04
	Truncated Normal	1.0110	0.44	0.0035	1.21E-05

Table B2 shows that the multiplication threshold remains unchanged for random distributions for 15% and 20% uncertainty, but changes for 25% uncertainty with normal, uniform, and truncated normal

References

1. Yilmaz, A., Acikgoz, H. & Elrouby, A. B. A. Building a surrogate model of a perfect electric conductor using polynomial chaos expansion and the characteristic mode analysis. *Turkish J. Electr. Eng. Comput. Sci.* **30**, (2022).
2. Nobile, F., Tempone, R. & Webster, C. G. A sparse grid stochastic collocation method for partial differential equations with random input data. *SIAM J. Numer. Anal.* **46**, (2008).
